# Characterization of protein expression of *Platanus* pollen following exposure to gaseous pollutants and vehicle exhaust particles

**DOI:** 10.1007/s10453-014-9327-5

**Published:** 2014-02-08

**Authors:** Senlin Lu, Jingjin Ren, Xiaojie Hao, Dingyu Liu, Rongci Zhang, Minghong Wu, Fei Yi, Jun Lin, Yonemochi Shinich, Qingyue Wang

**Affiliations:** 1 School of Environmental and Chemical Engineering, Shanghai University, Shanghai, 200444 China; 2 Shanghai Institute of Applied Physics, Chinese Academy of Sciences, Shanghai, 201800 China; 3 Center for Environmental Science in Saitama, Saitama, 374-0115 Japan; 4 School of Science and Engineering, Saitama University, Saitama, 338-8570 Japan

**Keywords:** *Platanus* pollen, Air pollution, Protein expression

## Abstract

Being major ornamental street trees, species of *Platanus* are widely planted in the Shanghai urban area. A great deal of allergenic *Platanus* pollen is released from the trees and suspended in the atmosphere during its flowering season, ultimately causing allergic respiratory diseases. Few papers have focused on the distribution of this type of pollen and its expression of allergenic proteins. In order to investigate any differences in protein expression in *Platanus* pollen following exposure to gaseous and particulate pollutants, a special apparatus was designed. Exposure condition (such as temperature, humidity, and exposure time) of *Platanus* pollen and gaseous pollutants can be simulated using of this apparatus. Fresh *Platanus orientalis* pollen, pollutant gases (NO_2_, SO_2_, NH_3_), and typical urban ambient particles (vehicle exhaust particles, VEPs) were mixed in this device to examine possible changes that might occur in ambient airborne urban pollen following exposure to such pollutants. Our results showed that the fresh *P. orientalis* pollen became swollen, and new kinds of particles could be found on the surface of the pollen grains after exposure to the pollutants. The results of SDS-PAGE showed that five protein bands with molecular weights of 17–19, 34, 61, 82, and 144 kDa, respectively, were detected and gray scale of these brands increased after the pollen exposure to gaseous pollutants. The two-dimensional gel electrophoresis analysis demonstrated that a *Platanus* pollen allergenic protein (Pla a1, with a molecular weight of 18 kDa) increased in abundance following exposure to pollutant gases and VEPs, implying that air pollutants may exacerbate the allergenicity of pollen.

## Introduction

Global warming has been cited as the cause of the increase in abundance of atmospheric pollen that has been observed over the last three decades (D’Amato et al. 2010), additionally, meteorological changes (such as, temperature, extreme weather events) induced by global warming have impacted the production, distribution, dispersion, and allergenic content of aeroallergens and the growth and the distribution of organisms that produce them (i.e., weeds, grasses, trees, and fungus) (Reid and Gamble 2009). Further, as the rapid growth of urban green areas continues, along with the number of plant species used, a corresponding increase in the rate of pollen allergies among patients has been reported (Riediker et al. 2001). Several studies have shown that the increasing incidence of pollen allergies has become a global problem (Ishizaki et al. 1978; Hwang et al. 2005; Bosch-Cano et al. 2011); for example, pollinosis has increasingly been found to have a profoundly negative effect on human health in Japan (Wang et al. 2011), in Europe (Fernandez-Gonzalez et al. 2010; Bosch-Cano et al. 2011), and in north America (Ziska et al. 2011). In China, the average incidence rate of hay fever is about 0.5–1 %; however, in particular areas, this figure can reach up to 5 %, such as in Wuhan, which is located in the center of China (Shi and Zhu 2009). The results of a survey conducted by our group also found that asthma caused by ambient pollen has led to 8.23 % of Shanghai children not being able to participate in outdoor physical activities and caused 5.02 % to be absent from educational services for more than two months (unpublished report). However, ambient particulate matter pollution remains at high levels in Shanghai (Lu et al. 2011). Our previous study demonstrated that the amount of ambient pollen and the atmospheric concentration of fine/ultrafine particulates is both important with respect to human health (Feng et al. 2011; Lu et al. 2011) and that pollen grains were one of the components in Shanghai atmospheric fine particulates (Lu et al. 2008). Furthermore, there were more fine particles observed on the surface of the allergenic pollen collected in urban areas compared with those collected in the suburban areas (Feng et al. 2011). Considering the fact that ambient pollen can be a carrier of various pollutants (atmospheric fine particulates, SO_x_, NO_x_, etc.) (Okuyama et al. 2007; Bellanger et al. 2012) and that species of *Platanus* are widely planted in parks and avenues in Shanghai, we hypothesized that *Platanus* pollen and air pollutants may have synergistic effects with regards to allergenicity and human health.

Several researchers have focused on the synergetic effects that may result from the interaction between air pollutants and pollen allergens. Parnia et al. (2002) suggested that traffic pollution generated ozone, nitrogen oxides (NO_x_), and inhalable particulate matter (PM_10_), and these were likely the atmospheric pollutants responsible for promoting hay fever. Okuyama et al. (2007) reported that airborne fine particulates were easily absorbed by pollen, and this interaction had a negative impact on human health. In addition, the pollen’s migration and precipitation into the nasal cavity and other parts of the respiratory tract, and it was argued that heavy metals and acidic substances contained in polluted pollen grains may exacerbate the occurrence of hay fever in urban residents. Chehregani and Kouhkan (2008) demonstrated that diesel vehicle emissions particles (DEPs) were capable of inducing pollen to produce new allergenic proteins.

Based on the literature cited above, we hypothesized *Platanus* pollen and air pollutants have synergistic effects. In order to testify our hypothesis, an exposure apparatus was designed to simulate the interaction between pollen and typical air pollutants. Our primary aim is to elucidate how environmental conditions likely contribute to the expression of allergenic proteins released by *Platanus* pollen.

## Materials and methods

### *Platanus* pollens sampling

Fresh *Platanus* pollen grains were sampled from *Platanus orientalis* tree trimmers (these trees located in the campus of East China Normal University). After branches with inflorescences were cut off the tree, the inflorescences were air-dried at room temperature and kept from any pollutants off. Pollen grains were collected from the dried inflorescences. The collected pollen was kept at −4 °C until subsequent use.

### Pollutants exposure apparatus

**Fig. 1:**
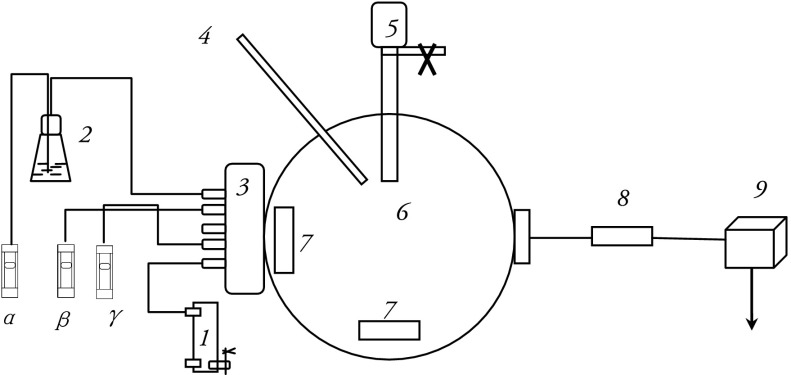
Diagram of the apparatus designed to simulate pollen exposure to air pollutants. SO_2_, NO_2_, and NH_3_ were transferred into the reactor, and the pollen was suspended in the reactor; after a set amount of time, pollen grains were collected according to the protocol. The apparatus contained the following components: 1-clean air; 2-gas-washing bottle; 3-mixer for gases; 4-sensor for temperature and humidity; 5-pollen; 6-chamber; 7-electronic fan; 8-sampler inlet head with filter; and 9-pump, α-SO_2_, β-NO_2_, γ-NH_3_

In order to investigate allergenic protein changes in *Platanus* pollen induced by air pollutants, an apparatus was designed to simulate the exposure of pollen to ambient urban gaseous air pollutants, including SO_2_, NO_2_, and NH_3_ (Shanghai Weichuang Standard Gas Analytical Co. Ltd.) as well as vehicle exhaust particles (VEPs, NIES, National Institute for Environmental Studies, Yatabe-Machi, Tsukuba, Japan). The exposure devices included an airflow meter, a humidification device, a mixer for gases, a temperature and humidity sensor, a pollen container, an exposure chamber, an electronic fan, a sampler inlet head with filter, and a low capacity pump (Fig. 1). The exposure chamber is the where the reaction between *Platanus* pollen and atmospheric pollutants (SO_2_, NO_2_, NH_3_) took place. The particle capture device was used to capture the exposure pollens with a polycarbonate membrane filter.

Pollen grains were suspended by electronic fans in the chamber after SO_2_ (0.1 L/min), NO_2_ (0.54 L/min), NH_3_ (0.03 L/min) were transferred into the chamber. The mole ratio of SO_2_, NO_2_, and NH_3_ was kept 30:200:30 in the chamber (Yamamoto et al. 1995; Wang, unpublished report 2012), the mass concentration of the gaseous pollutants was 5–6 times higher compared with their measured mass levels in the air (Shanghai Environmental Bulletin claimed that mass levels of SO_2_ and NO_2_ were 23 and 46 μg/m^3^, respectively (SEPB 2012), while the humidity and temperature in the inner environment of the chamber were kept at 60 % and 25 °C, respectively (which are similar to the values seen during pollen season).

### Scanning electron microscopy (SEM) analysis

A very small amount of pollen was put on a double-sided adhesive and conductive carbon tape with a clean wooden pick. Following this step, gentle blowing was applied so as to remove loosely stuck pollens from the carbon tape. The remaining pollen was examined by SEM.

After the carbon tape was coated with gold, the sample was observed under a scanning electron microscope (Zesis ultra 55 FE-SEM; Germany) with the following parameters: voltage, 30 kV; resolution, 3 nm; and scanning distance, 15.0 mm. The chemical composition on the pollen was investigated by X-ray energy dispersive spectroscopy (EDX) (UK 7421, Oxford).The EDX spectrometer was Link ISIS spectrometer with a Si(Li) detector, which allows X-ray detection from elements higher than carbonate (*Z* > 6) (Lu et al. 2006).

### Protein analysis

Sodium dodecyl sulfate polyacrylamide gel electrophoresis (SDS-PAGE) was used for protein analysis. A modified version of the pollen protein extraction protocol described by Varela et al. (1997) was used in this study. Briefly, 2 g of fresh *Platanus* pollen was defatted with acetone (3.44 mol/L), dried, and extracted in 40 ml of 0.01 M phosphate and 0.15 M NaCl phosphate-buffered saline (PBS; pH = 8); the mixture (1:20 wt/vol) was stirred for 12 h at 4 °C. The suspension was filtered through a cellulose filter paper (Whatman Ltd., Maidstone, UK), dialyzed against PBS, and sterilized by 0.22 μm filtration. The total protein content of the pollen extracts was determined by the Bradford protein assay using bovine serum albumin (BSA) as the standard. Extracted proteins were separated by 10 % SDS-polyacrylamide gel electrophoresis (90 μl of each pollen extract per well) and visualized by Coomassie blue staining.

### Analysis of protein expression by two-dimensional gel electrophoresis (2-DE)

The two-dimensional gel electrophoresis (2-DE) protocol was as described by Sheoran et al. (2009). An equal amount of protein (200 μg) sample was mixed with fresh rehydration buffer (9 M Urea, 4 % CHAPS, 1 % DTT, 1 % IPG buffer, and trace amounts of bromophenol blue) to a total volume of 450 μL. The protein samples were then added to the strip holder. The isoelectric focusing protocol was carried out at 20 °C, with 50 μA per strip. The strips were laid across the top of the gel, making sure that the gels were lying flush with one another. After the sealing solution cooled and solidified, the strip was moved to the electrophoresis apparatus (Ettan-DALT-Six system; GE healthcare; USA). Electrophoresis was carried out at 15 °C, and the gel was run at 100 V for 45 min, followed by 200 V for 6–8 h (until the bromophenol blue band reached the bottom of gel). The gel was visualized by silver staining as described previously (Shevchenko et al. 1996). All gel images were processed by a three step procedure: (1) protein spot detection; (2) quantify volume of the selected spot; and (3) match grayscale values using Bio-Rad PDQuest 8.0 software.

### *Platanus* protein identification with mass spectrometry (MS)

*Platanus* protein identification by mass spectrometry (MS) was carried out using the protocol described by Sheoran et al. (2009). Briefly, excised protein spots were automatically destained, dehydrated, reduced with DTT, alkylated with iodoacetamide, and digested with trypsin with a MassPREP protein digest station (Wates/Micromass; Manchester, UK). Samples were suspended in 5 μL 0.1 % TFA, followed by mixing (in 1:1 ratio) with a matrix consisting of a saturated solution of α-cyano-4-hydroxy-*trans*-cinnamic acid in 50 % ACN and 0.1 % TFA. The 1 μL mixture was spotted on a stainless steel sample target plate. Peptide MS and MS/MS were performed on an MALDI-TOF/TOF (AB SCIEX; USA) plus mass spectrometer. Data were acquired in a positive MS reflector using a CalMix5 standard to calibrate the instrument. Both the MS and MS/MS data were integrated and processed with the use of the GPS Explorer v3.6 software with default parameters. Employing the combined MS and MS/MS spectra, proteins were successfully identified based on 95 % or higher confidence interval of their scores in the MASCOT v2.1 search engine (Matrix Science Ltd.; London, UK).

## Results

### Microscopic characterization of ambient pollen

**Fig. 2:**
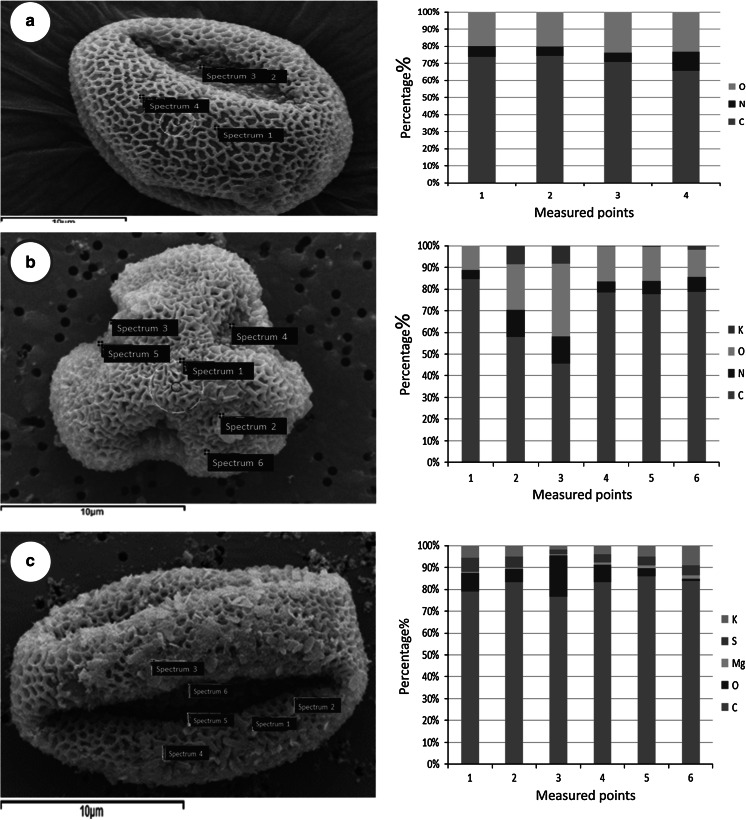
Microscopic images of the *Platanus orientalis* pollen before and after exposure to air pollutants. **a** Fresh pollen before exposure; **b** pollen after 1 h exposure; **c** pollen after 8 h exposure. The corresponding EDS analysis of every time point is displayed on the left

Results of SEM analysis revealed that the *P. orientalis* pollen grains were suboblate, with a diameter 20–30 μm, and with trenches on its surface. The surface of fresh pollen grains became swollen after exposure to the mixture of gases. Further, EDX spectral analysis demonstrated that C, N, and O were the main elements on the surface of pollen before exposure to the air pollutants (Fig 2a). After 1 h exposure, new particles could be found on the surface (Fig 2b), and these were composed of K, O, N, and C. The number of the new particles increased with exposure time, and S could be detected in the new particles formed after 8 h exposure (Fig 2c). The formation of these new particles suggested that a chemical reaction occurred during the contact of the pollen with the gases.

**Fig. 3:**
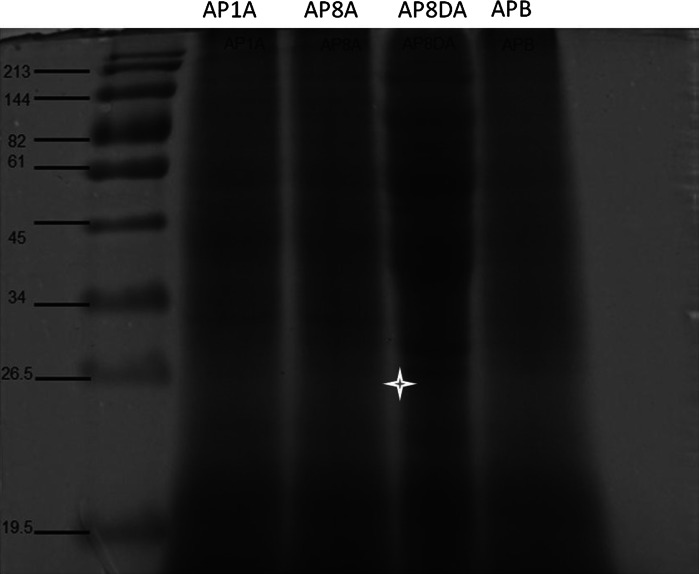
Protein profile of *Platanus* pollen extracts. Major protein bands were detected, and a new band (26.5 kDa) was found in AP8DA (indicated by star). AP1A and AP8A refer to pollen exposed to pollutant gases for one and 8 h, respectively. AP8DA refers to pollen exposed to pollutant gases and VEPs for 8 h. APB refers to pollen before exposure to pollutants

### Separation of total protein by SDS-PAGE

The results of SDS-PAGE analysis showed that protein bands with molecular weights of 17–19, 34, 61, 82, and 144 kDa could be found in samples of fresh pure *Platanus* pollen (herein thereafter named as APB). After pollen exposure to gaseous pollutants for 1, and 8 h, the main protein bands became dark, suggesting more protein was expressed by the pollen grains. In this study, a new protein band was found after the exposure of pollen to gaseous pollutants and VEPs for 8 h. The presence of the new protein band, with an approximate molecular weight of 26.5 kDa, implies that VEPs stimulate fresh *Platanus* pollen to express new proteins.

### Differences in protein expression identified by two-dimensional gel electrophoresis (2-DE)

**Fig. 4:**
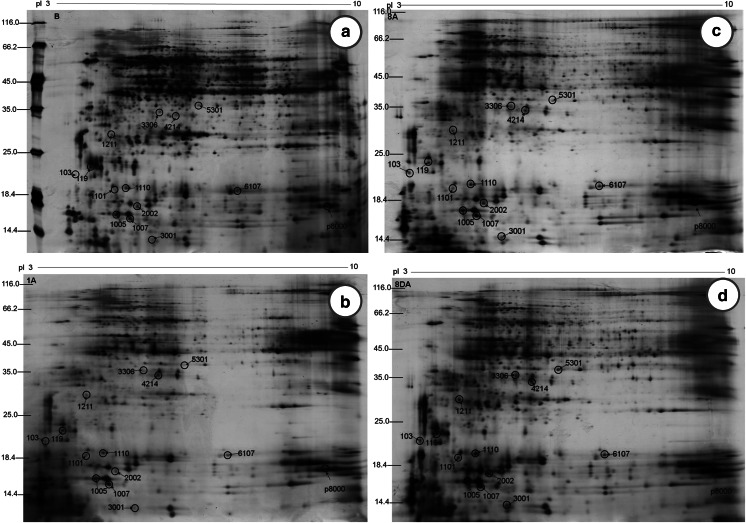
Two-dimensional gels of *Platanus* pollen protein extracts. Molecular masses are given in kDa, and the approximate isoelectric points are shown. **a** APB; **b** AP1A; **c** AP8A; and **d** AP8DA

The 2-DE assay has been widely used to distinguish differences between complex protein extracts. On the basis of a range of biochemical properties, including charge (pI), size (M), and hydrophobicity, proteins can separated from one another with this technique. Figure 4 depicts the protein spots showing changes after the exposure of *P. orientalis* pollen to air pollutants. Grayscale values (listed in Table 1) of 14 protein spots, p103, p119, p1005, p1007, p1101, p1110, p1211, p2002, p3001, p3306, p4214, p5301, p6107, and p8000, were found to increase following different exposure conditions. It was worth to note that grayscale value of p1007 increased more than 4 times after the pollen exposure to gaseous pollutants and VEPs for 8 h. The protein spot (p8000) with molecular weight ~18 kDa and pI ~9.3 was identified as Pla a1 according to the literature (Asturias et al. 2002).

**Table 1:** Variation in the grayscale values of protein spots

Spot no.	APB	AP1A	AP8A	AP8DA
p103	20.84	479.03	3,149.84	3,701.75
p119	1,524.36	1,911.98	2,784.16	3,222.32
p1005	9,339.34	11,145.98	16,187.35	17,450.83
p1007	2,848.85	4,783.29	10,353.78	12,698.74
p1101	20.13	371.25	2,147.11	2,181.65
p1110	2,156.73	4,910.28	5,157.82	6,720.15
p1211	582.93	719.38	1,549.03	4,104.42
p2002	2,116.08	2,856.54	5,757.24	5,935.31
p3001	119.85	120.29	3,907.27	4,523.72
p3306	81.51	142.65	2,194.38	3,627.75
p4214	63.45	3,511.27	3,711.09	4,521.69
p5301	50.28	53.76	1,427.74	3,220.31
p6107	2,156.04	2,388.99	4,618.66	4,743.19
p8000	4,576.98	7,812.28	6,563.98	16,754.91

### MS analysis

**Fig. 5:**
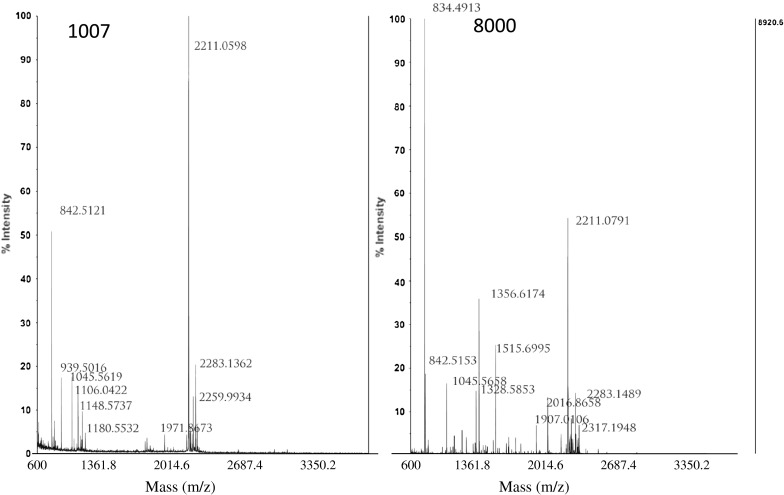
TOF-MS spectra of spot 1007 and spot 8000

Two protein spots (p1007 and p8000) in the 2-DE gel were selected to identify their protein constitution using of mass spectrometry (Fig. 5).

According to Mascot Score Histogram, four proteins were found in spot p1007 (Table 2): gi|12229949 (RecName: Full=40S ribosomal protein S12); gi|195604208 (40S ribosomal protein S12 [Zea mays]); gi|226505142 (ribosomal protein, S12 (homolog) [*Zea mays*]); and gi|116782336 (unknown [*Picea sitchensis*]). None of these proteins are a documented allergenic protein. Only one protein was found in protein spot p8000: gi|29839547 (RecName: Full=Putative invertase inhibitor; AltName: Full=Pollen allergen Pla a 1; AltName: Allergen=Pla a 1; Flags: Precursor). The amino acid sequence of the Pla a 1 is: MKLSFSLCIF FFNLLLLLQA VISADIVQGT CKKVAQRSPN VNYDFCVKSL GADPKSHTAD LQGLGVISAN LAIQHGSKIQ TFIGRILKSK VDPALKKYLN 101DCVGLYADAK SSVQEAIADF KSKDYASANV KMSAALDDSV TCEDGFKEKK GIVSPVTKEN KDYVQLTAIS LAITKLLGA. This amino acid sequence further demonstrated the identity of the allergenic protein expressed by the *Platanus* pollen.

**Table 2:** Identification of proteins expressed in *Platanus orientalis* pollen following exposure to gaseous pollutants

Spot no.	Gene index	Protein identity(TAIR description)	MM/pI^a^	Mascot score	matches	Sequence coverage
1007	gi|12229949	RecName: Full=40S ribosomal protein S12	15,627/5.35	102	2(1)	23
	gi|195604208	40S ribosomal protein S12 [Zea mays]	15,361/5.33	71	1(1)	12
	gi|226505142	ribosomal proteinS12 (homolog) [Zea mays]	15,326/5.51	71	1(1)	12
	gi|116782336	unknown [Picea sitchensis]	13,331/6.19	64	1(1)	7
8000	gi|29839547	RecName: Full=Putative invertase inhibitor; AltName: Full=Pollen allergen Pla a 1; AltName: Allergen=Pla a 1; Flags: Precursor	19,555/8.89	172	3(3)	26

^a^Molecular mass (Da) and pI of identified protein (according to the Proteinscape software; Bruker Daltonics)

^b^Mascot score (http://www.matrixscience.com/help/scoring_help.html)

^c^Sequence coverage (percentage of the complete protein sequence identified)

## Discussion

*Platanus* trees are widely grown as street and shade trees around cities in the world. This kind of tree includes *Platanus occidentalis, Platanus orientalis,* and *Platanus acerifolia*. Our field survey results showed that most of *Platanus* tree is widely planted in parks and avenues in Shanghai is *P. orientalis*. And this fielding survey results agreed with our microscopic characterization results, which the *Platanus* pollen collected in Shanghai was the same as *P. orientalis* pollen provided by GREER (Lenoir, NC, USA) (data not shown).

Previous study has indicated that *Platanus* pollen is a major contributor to pollinosis symptoms during March and April. Extensive studies on *P. acerifolia* allergy have been reported, however, there are few reports on allergenic protein released by *P. orientalis* trees (Pazouki et al. 2009).

Varela et al. (1997) reported *Platanus* pollens (including *P. orientalis* and *P. acerifolia*) have been tested to contribute to the symptoms of patients with pollinosis. *Platanus* pollen in Shanghai urban atmosphere is obviously greater abundance compared with that in the suburban and rural area during its flowering season (data not shown). Importantly, main air pollutants in urban atmosphere (such as atmospheric fine particulates, sulfates, SO_x_, NO_x_), could be absorbed on the surface of *Platanus* pollen (Feng et al. 2011). These different particle types not only reflected their different sources, but would also enhance allergenicity through different mechanisms (D’Amato et al. 2007). Considering pollen allergen liberation could be affected by air pollutants (Behrendt et al. 1997; Majd et al. 2004), we hypothesized that allergenic protein expression released from *Platanus* pollen might be affected by air pollutants.

Our exposure experiment results showed that pollen grains become swollen and new particles could be found on the pollen surface after exposure to the mixture of gases. Chehregani et al. (2004) also demonstrated that pollen grains became folded, and airborne particles accumulate on the surface of *Zinnia* pollen, after grains were exposed to the polluted air of Tehran for 20 days. The morphological change of ambient pollen might affect behavior of its allergenic contents (Wang et al. 2011).

Protein released from *P.* *acerifolia* pollen has been studied, three allergenic *P. acerifolia* pollen proteins have been identified: one minor allergen (Pla a 3) (an aeroallergen related to food allergy) and two major allergens (Pla a 1 and Pla a 2), with molecular weights of ~18 and ~44 kDa, respectively (Asturias et al. 2002, 2003; Lauer et al. 2007). Pla a1 is a non-glycosylated protein, while Pla a2 is a glycoprotein (Ibarrola et al. 2004; Fernandez-Gonzalez et al. 2010). Pla a1 represents ~60 % of the total IgE binding of *Platanus* pollen extract and can be used for specific diagnosis of *Platanus* (*orientalis* and *acerifolia*) pollen allergy (Asturias et al. 2003; Pazouki et al. 2009). Therefore, investigation of proteins and identification of Pla a1 released from *Platanus* pollen planted in Shanghai could provide fundamental data for evaluation of health risks caused by this kind of ambient allergenic pollen.

Our SDS-PAGE results demonstrated that there existed protein with molecular weights of 17–19 k Da in the *Platanus* pollen, and its graylevels increased after pollen exposure to air pollutants, suggesting more protein released. Varela et al. (1997) reported major protein bands of 17, 43, 45 kDa from *Platanus* *(acerifolia)* pollen, and suggested that the 17 kDa band was the allergenic protein. Asturias et al. (2006) claimed the presence of several allergens in *Platanus (acerifolia)* extracts, but only two of them, Pla a 1(18 kDa) and a 43 kDa were allergenic protein. While in China, several studies focused on the identification of proteins from *Platanus* pollen, for example, Li et al. (2004)found 6 major protein bands with *Platanus* pollen (16, 22, 35, 39 and 71 kDa) and claimed that those 22–71 kDa in size were the major allergenic proteins based on Western blot analysis. It must be pointed out that *Platanus* pollen was defatted with acetone (3.44 mol/L) in the procedure of protein extracts in this study, and this chemical regent might affect the quantity and quality of allergenic content which accumulated in the intine (Suarez-Cervera et al. 1995), more studies are needed to investigate the negative effects on allergenic protein extracts in future work.

In order to testify the protein with molecular weights of 17–19 k Da in the *Platanus* pollen and its variety after the pollen exposure to the gaseous pollutants, two-dimensional gel electrophoresis (2-DE) and mass spectrometry were employed. The 2-DE result clearly demonstrated grayscale values of 14 protein spots (Table 1) were found to increase, following our different exposure conditions, implying protein expression in *Platanus* pollen increase with exposure time and with SO_2_, NO_2_, NH_3_, and VEPs. Among the 14 protein spots, p1007 and p8000 were selected for further study by mass spectrometry (Sheoran et al. 2009). The p1007 contained 4 kinds of protein, in which none of them was allergenic and only one protein was found in the p8000 protein spot from the NCBInr database (Table 2). This protein was clearly identified as Pla a1 according to its amino acid sequence.

Behrendt et al. (1997) reported that air pollutants were one of inducers of allergen liberation, and Chehregani and Kouhkan (2008) argued that new allergenic proteins could have been found after *Lilium martagon* pollen exposure to diesel vehicle emissions particles (DEPs). A new protein band, with an approximate molecular weight of 26.5 kDa, also was found after *Platanus* pollen exposure to VEPs in this study. Therefore, our results might demonstrate that air pollutants could affect allergen liberation from *Platanus* pollen. Further experiments dissecting this phenomenon will be needed.

Among allergenic proteins from *Platanus* pollen, Pla a1 (18 kDa protein) was responsible for 79 % of the Ig-E binding capacity and could be as a reliable diagnosis of *Platanus* (*acerifolia*) pollen in the allergenic reaction (Asturias et al. 2006), and was characterized using of immunoglobulin (Ig) E-immunublot analysis (Asturias et al. 2002). Based on the previous literature, the Pla a1 of *P.* *orientalis* pollen was regarded as a unique allergen (Asturias et al. 2003; Pazouki et al. 2009), it is reasonable to deduce that the Pla a1 protein expression increased after the *P.* *orientalis* pollens exposure to air pollutants, and it will contribute to pollinosis symptoms.

## Conclusions

A special apparatus was designed to investigate any differences in protein expression in *P.* *orientalis* pollen after exposure to air pollutants, the following results were achieved: (1) after *P.* *orientalis* pollen exposure to the pollutant gases and particles, the pollen became swollen, and new kinds of particles could be found on the surface of the grains; (2) the results of two-dimensional gel electrophoresis and mass spectrometry analysis demonstrated that the expression of an allergenic protein (Pl a a1) of *P.* *orientalis* pollen is increased after exposure to pollutant gases and VEPs, implying that air pollutants can exacerbate the allergenicity of pollen of this genus.
